# Autofluorescence imaging for improved visualization of joint structures during arthroscopic surgery

**DOI:** 10.1186/s40634-017-0094-4

**Published:** 2017-06-02

**Authors:** Duy Tan Nguyen, Pepijn van Horssen, Hans Derriks, Martijn van de Giessen, Ton van Leeuwen

**Affiliations:** 1 Present Address: Department of Family Medicine, University of Gent, Ghent, Belgium; 2 Department of Orthopaedic Surgery, University of Amsterdam, Amsterdam, The Netherlands; 3 Department of Physics and Medical Technology, VU University Medical Center, Amsterdam, The Netherlands; 4 Biomedical Engineering and Physics, University of Amsterdam, Amsterdam, The Netherlands; 5 Present Address: St. Maartenskliniek, Nijmegen, The Netherlands; 6 Department of Radiology, Leiden University Medical Center, Leiden, The Netherlands; 7 Department of Intelligent Systems, Faculty of Electrical Engineering, Applied Mathematics and Computer Science, Delft University of Technology, Delft, The Netherlands

**Keywords:** Arthroscopy, Fluorescence arthroscope, Arthroscopic autofluorescence imaging system, Anterior cruciate ligament, Fluorescence

## Abstract

**Background:**

The purpose of our study is to develop the arthroscopic autofluorescence imaging (AFI) system to improve the visualization during arthroscopic surgery by real-time enhancing the contrast between joint structures with autofluorescence imaging. Its validity was evaluated around the arthroscopic anterior cruciate ligament (ACL) reconstruction, specifically improving the contrast between the femoral insertion site and its background. The feasibility of the AFI system was validated with bovine and human knees. The spectral responses of the femoral insertion site and its surrounding bone and cartilage were measured with a fluorospectrometer. A prototype of the AFI system was developed based on the spectral responses (SR) and test images of the insertion site. The accuracy was validated by evaluating the overlap between manually segmented insertion sites on the white light color images and on the corresponding spectral unmixed autofluorescence images. The final prototype of the AFI system was tested during arthroscopy in cadaveric knees.

**Results:**

The results showed that the joint structures have different SRs. Spectral unmixing enabled separation of the SRs and improved the contrast between the joint structures. The agreement between visible light and autofluorescence ligament insertions had a mean Dice coefficient of 0.84 and the mean Dice coefficient of the interobserver variability for visible light imaging was 0.85.

**Conclusions:**

We have shown that the femoral insertion site can be accurately visualized with autofluorescence imaging combined with spectral unmixing. The AFI system demonstrates the feasibility of real-time and subject-specific visualization of the femoral insertion site which can facilitate anatomic ACL reconstruction. In addition, the AFI system can facilitate arthroscopic procedures in other joints and can also be used as a diagnostic tool.

**Electronic supplementary material:**

The online version of this article (doi:10.1186/s40634-017-0094-4) contains supplementary material, which is available to authorized users.

## Background

Many joint surgeries are nowadays performed arthroscopically. This minimally invasive approach has resulted in a reduction of post-operative complications, faster recovery and improved patient outcome compared to open surgery (Watanabe 1972; Strobel 2002; Miller and Cole 2004; Scuderi et al. 2010). Accordingly, numerous arthroscopic procedures for small and large joints have been developed. Currently, more than 4 million arthroscopic procedures in the knee joint are performed worldwide per annum and this number is increasing (Solomon 2008). Despite its advantages, arthroscopic joint surgery is challenging due to the restricted working space, hand-eye coordination, lack of palpation and visual limitations. The visual limitations are related to the standard white light endoscopic systems and include: small viewing angle and poor contrast between the collagenous joint structures (as they all appear off-white). Additionally, injury or degeneration of the joint structures further hampers the contrast and visualization (Strobel 2002). Thus, improved contrast between the joint structures will be of great value as clear visual feedback is paramount for effective and safe arthroscopic surgery. Recent advances in optical imaging demonstrated the importance of fluorescence imaging in the field of urology, gastroenterology and abdominal surgery (Nguyen and Tsien 2013; Vahrmeijer et al. 2013). Researchers were able to visualize precancerous tissues, tumors and metastasis in real-time and with high contrast by using exogenous fluorescent markers against the targeted structures. This approach facilitated more complete tumor resection and even resection of (pre-)malignant tissues that otherwise were not detected with the standard white-light endoscopy. Similarly, the autofluorescence of tissue components can be utilized to enhance the visibility of tissues with low contrast under regular visible light reflectance imaging. Collagen is such an autofluorescent tissue component which can emit blue/green light (emission 400–405 nm) after excitation with violet/blue light at (325 nm). The autofluorescence of collagen is associated with cross-links, hydroxylysyl pyridoline and lysyl pyridinoline. (Fujimoto et al. 1978; Eyre et al. 1984) The imaging of the collagen's autofluorescence may be advantageous for orthopedic surgeons and rheumatologists as collagen is the main constituent of the joint structures such as ligaments, bone and cartilage.

Arthroscopic anterior cruciate ligament (ACL) reconstruction is one of the most performed surgical procedures by the orthopedic surgeon. The location of the bone tunnels is a crucial factor for the success of the reconstruction surgery and the bone tunnels should be placed at the original insertion site of the ruptured ACL (van Eck et al. 2010; Chechik et al. 2013). Hence, graft misplacement is the most common cause of revision surgery (Greis et al. 1993; Sommer et al. 2000; Trojani et al. 2011; Morgan et al. 2012; Rahr-Wagner et al. 2013) and small deviations can result in large changes in knee stability (Howell and Taylor 1993; Markolf et al. 2002; Loh et al. 2003). Before the introduction of arthroscopic surgery, the surgeon could easily distinguish the insertion sites from the surrounding bone and cartilage due to the fact that the whole knee joint was exposed. With the introduction of the arthroscope, the localization of the insertion site became more challenging due to the aforementioned technical and visual limitations. Our research question was whether the femoral insertion site can be accurately visualized with autofluorescence imaging either with or without image post-processing techniques such as spectral unmixing. The purpose of our study is to develop the arthroscopic autofluorescence imaging (AFI) system to improve the visualization during arthroscopic surgery by real-time enhancing the contrast between joint structures with autofluorescence imaging. Its validity was evaluated around the arthroscopic anterior cruciate ligament (ACL) reconstruction, specifically improving the contrast between the femoral insertion site and its background. We investigated three different approaches to visualize the ACL insertion site, each one more directed to a practical implementation of a clinical imaging setup. First, we explore the possibility to maximize the contrast of the ACL with its surroundings by choice of optimal excitation and emission wavelengths such that a unique ACL insertion site image is obtained. Secondly, we apply a post-processing strategy where a fixed excitation wavelength is used for illumination with spectral unmixing performed on two different emission images obtained consecutively. And third, a real-time approach is shown which utilizes the spectral unmixing principal on separate color channels of RGB images with a fixed excitation wavelength. The optimal set of imaging parameters was determined to develop a pre-clinical prototype of AFI system to improve the contrast between joint structures during arthroscopy, particularly between the insertion site with respect to bone and cartilage. The boundary conditions for autofluorescence arthroscopy (AA) we imposed were accuracy, real-time, robust, and compatible with the current white light endoscopic systems. We evaluated our approach on cadaveric bovine and cadaveric human knees. We hypothesize that the spectral response between these tissues differs and thus the femoral insertion site can be visualized separately based on the difference in the spectral response due to the difference in biochemical composition, and specifically in collagen content and collagen distribution.

## Methods

### Method 1 and 2: Spectral responses of bovine and human ACL insertion sites, bone and cartilage

Fresh bovine cadaveric knees (*n* = 10) from a local abattoir (Van Kooten B.V., Montfoort, The Netherlands) were prepared for the excitation and emission scans. The knees were all dissected until the ACL was exposed. The ACL was removed with an arthroscopic scissor punch (Arthrex, Naples, USA), mimicking the clinical situation, and leaving a short remnant (±2 mm) on the lateral wall of the intercondylar notch. The bovine samples were placed in a light blocking box on a height adjustable table. The optic fiber of the fluorospectrometer (Perkin-Elmer, LS55, Rodgau, Germany) was consecutively aimed at the femoral insertion of the ACL, its bony surroundings on the lateral condyle and the cartilage of the lateral femoral condyle. A 5 mm thick reference was placed between the sample and the optical fiber probe and subsequently the z-axis table was adjusted until the distance between the sample and the probe was 5 mm. Excitation-emission maps of the samples were obtained by scanning the excitation wavelength, starting at 280 nm and ending at 480 nm in steps of 10 nm, while detecting the emission light from 300–800 nm. The excitation and emission slits used during the experiment were both 2.5 nm. During all the experiments dehydration of the samples was prevented by moistening them with 0.9% NaCl solution. Fresh-frozen human cadaveric knees (*n* = 3) were thawed and cut into three small samples (5 mm x 5 mm x 8 mm) each containing only the insertion site, bony surrounding and cartilage and placed consecutively in the cuvette holder of the fluorospectrometer. Again, excitation emission maps were obtained (excitation wavelength from 200 nm to 490 nm in 10 nm steps and an emission range of 200–700 nm). After obtaining the excitation and emission spectra, an optimal combination of excitation and emission wavelengths to visualize the insertion site was determined by calculating the difference in intensity and difference between emission peaks.

### Method 3: ACL insertion site imaging based on spectral unmixing

Tissue autofluorescence spectra tend to be wide and strongly overlapping (Wagnieres et al. 1998). Therefore, obtaining a set of excitation and emission wavelengths that can directly separate tissues visually is not possible in general. However, using wavelength dependent ratios between tissue specific spectra, imaged tissues can be separated (at 520 nm: ACL/Bone = 1.56 (0.31 SD) and at 620 nm: ACL/Bone = 1.05 (0.16 SD), material and set-up dependent). This method, called spectral unmixing, requires at least as many images as the number of structures to be separated (Keshava and Mustard 2002). We used a straightforward two component linear spectral unmixing technique, with the components identified as ACL insertion site (520 nm ± 7 nm) and surrounding tissue composed primarily of bone (620 nm ± 7 nm). They have to be extracted only once from fluorescent images by manually measuring the ratio of intensity of the ACL and bone for both images excited at 520 and 620 nm respectively. These factors can then be used to do spectral unmixing for all other data sets obtained with that particular imaging setup. The excitation wavelength is chosen such that a maximum difference between the ratio of the components exists in the emission images, as this provides the conditions for maximal contrast between ACL and it surroundings (Keshava and Mustard 2002).

### Validation of spectral unmixing in bovine knees

As stated above, many open joint surgeries became outdated with the introduction of arthroscopic surgery. At the same time, visualization with arthroscopic surgery is much more difficult than open surgery. To evaluate whether arthroscopic surgery combined with autofluorescence imaging is useful, it is important to evaluate whether the borders of the ACL insertion visualized with autofluorescence imaging and open surgery view is comparable. If it is similar, we can conclude that the borders seen with the AFI system (autofluorescence imaging combined with a current arthroscopy system), are indeed the true borders of the ACL insertion. In other words, due to the fact that the ACL insertion site can easily be seen in open knees with an open view, the open view was used as the gold standard to evaluate the accuracy of autofluorescence imaging by comparing the annotations of the femoral insertion site made in images acquired in white light of open dissected knees with the annotations acquired with fluorescence imaging in the same knees. The lateral condyle of 10 bovine knees was imaged with a hyperspectral imaging setup. Each sample was illuminated with five power-LEDs, with the optimal excitation wavelength derived from the fluorospectrometer measurements (Roithner lasertechnik, Vienna, Austria, H2A1-H395, 395 nm Roithner LaserTechnik GMBH 95 mW). Spectra were acquired by a hyperspectral camera equipped with a liquid crystal tunable filter (Varispec, PerkinElmer, Massachusetts, USA) and a digital camera (PCO.Pixelfly, Kelheim, Germany). The spectral sweep ranged from 400 nm to 720 nm with 2 nm steps and a spectral resolution of 7 nm. Spectra for ligament and bone were determined from annotations in one knee and measuring the intensity within the annotation for the full spectral range. Based on these spectra, two optimal emission images were selected with the largest difference in intensity ratio between ACL and its surroundings. The ratios between autofluorescence in the red and green channels were determined for both tissue types as intensity ACL/Bone = 2.20 in the red channel and intensity ACL/Bone = 1.89 in the green channel. The same light source and endoscope were used on the other samples, leaving difference between knee samples as only changed variable. Spectral unmixing was applied and the resulting visualization of ligament and bone were false colored in red and green, respectively. White light images were approximated by taking the average intensity of the images from: 470-500 nm for the blue channel, 510-560 nm for the green channel and 600–700 nm for the red channel. Three independent observers (M.F., T.N., and H.T.) drew contours of the femoral insertion site in the visible light and the spectrally unmixed images. The areas contained in these annotations were compared per observer between the unmixed and white light images. Again, as the femoral insertion site in an open knee is easily distinguished from the surrounding, the contours of the femoral insertion site in visible light is considered as the ground truth. The annotation accuracy was quantified by calculating Dice coefficient of the overlap between these annotations as D = 2(A⋃B)/(A + B), where A⋃B is the number of overlapping pixels in the segmented insertion sites and A + B is the total number of pixel in both insertion sites. D = 1 corresponds to a complete agreement and D = 0 signifies no agreement at all. The interobserver variability was evaluated for the annotations in unmixed and white light images separately, also by computing Dice coefficients, where consensus annotations were obtained by majority voting over the observers.

### Real-time spectral unmixing with prototype of Autofluorescence Arthroscope

Real-time imaging is a necessity for a clinical implementation, which requires simultaneous acquisition of the channels that capture the emitted light. The collagen autofluorescence spectrum spans the blue, red and green channel or regular white light endoscopy RGB cameras and consumer RGB cameras, allowing for the rapid building of a prototype. A real-time autofluorescence arthroscope was assembled which consisted of: 1) an external Light-Emitting-Diodes 390 ± 5 nm light source [OSA OPTO LIGHT, Berlin, Germany, OCU-400 UE390 OSA Opto light : 2.8–8.4 mW] to excite the collagen, 2) a 1.3 Mpixel digital camera Webcam Pro WB-6250X (Trust, Dordrecht, The Netherlands), 3) a 430 nm long pass filter to reflected excitation light, 4) a laptop, 5) custom image processing software written in Labview (Austin, Texas). The real-time video stream was processed using a real-time spectral unmixing of the red and green channels. Real-time video captures were made of the insertion sites in dissected bovine knees (*n* = 5) (see Additional file 1).

### Feasibility in human knees: ex vivo Autofluorescence Arthroscopy

The AA was combined with a commercially available endoscopy system (Smith & Nephew C.V., Hoofddorp, The Netherlands) and an autofluorescence arthroscopy was performed in fresh-frozen human knees (*n* = 2). The ratios of the intensity of ACL and bone, utilized for the spectral unmixing routine, are characteristic of the imaging setup used. They are dependent on the optical sensitivity of the system to the fluorescent emission spectra. The ratios for this setup, composed of the commercially endoscopic system, were extracted from the red and green channel of an RGB image of one human knee acquisition and used for unmixing the remaining knees. The video stream from the commercially available endoscopy system does not permit real-time separation of the RGB channels for unmixing, therefore the color video was saved and post-processed. Lateral and medial portals were made *lege artis*. The knee was irrigated with NaCl 0,9% (Baxter, Deerfield, USA) and routinely inspected with the arthroscope on the white light modus. The ACL was then shaved with a shaver blade (Dyonics, 4.5 mm Synovator), leaving a small remnant of the ACL of approximately 2 mm on the lateral femoral wall. The LED probe was then introduced to the knee joint and the FA was turned on.

#### Statistical test

An unpaired Student’s *t*-test was used for statistical analysis of the Dice’s coefficients. A probability of *p* < 0.05 was considered statistically significant.

## Results

### Spectral response of the bovine ACL insertion site, bone and cartilage

**Fig. 1:**
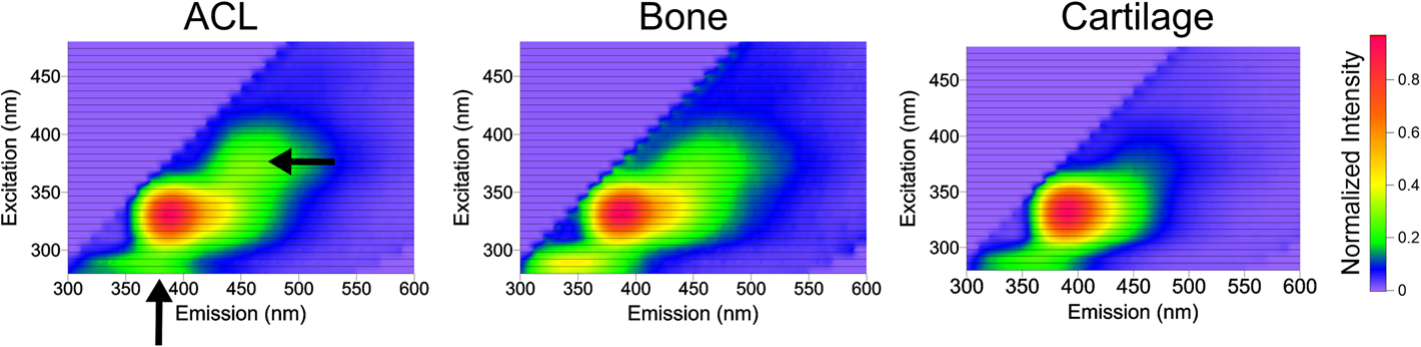
Typical 3D excitation-emission maps normalized to the peak intensity of insertion site (ACL), bone and cartilage from one bovine knee. Note the differences in autofluorescent response between the separate components indicated by arrows

Typical 3D excitation-emission maps of the three tissues within one bovine knee depict differences in intensity and shape (Fig. 1). Results throughout the study will be presented in arbitrary units (arb. units) and will be denoted in the figures as intensity values normalized to the peak value. For the insertion site (*n* = 10 bovine knees), the mean peak autofluorescence intensity was found at the emission wavelength 390 nm and at the corresponding excitation wavelength 330 nm. The autofluorescence intensity from the surrounding bone was measured at the same emission and excitation wavelength as the insertion site. The peak autofluorescence intensity from the surrounding cartilage was higher than the insertion site, and found at the emission wavelength 390 nm and the corresponding excitation wavelength 330 nm. The difference in intensity between the insertion and bone was 111 arb. units (interquartile range 54.6–180.1 arb. units). The difference in intensity between the insertion and the cartilage was −179.4 arb. units (interquartile range −150.2 - -220.7 arb. units).

The largest difference between the emission peaks of the insertion and the bone was 25 nm (interquartile range 20.25–33 nm) and found at an excitation wavelength of 280 nm. At this excitation wavelength the distance between the peaks of the insertion and the cartilage at the excitation was 8 nm (interquartile range −1.25–13.63 nm). For the insertion and the cartilage tissue, the largest difference was 44.5 nm (interquartile range 38.63–48.38 nm) and found at an excitation wavelength of 360 nm. At this excitation wavelength the distance between the peaks of the insertion and the bone was 3.5 nm (interquartile range −4.36 - -8.75 nm).

### Spectral response of human ACL insertion site, bone and cartilage

**Fig. 2:**
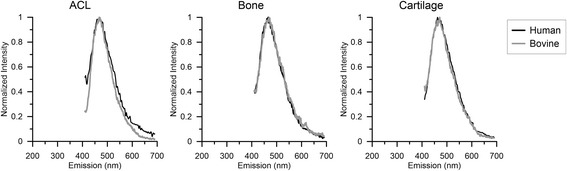
Emission spectra at 390 nm excitation of bone, ACL, and cartilage for the bovine (*gray*) and human (*black*) samples superimposed to display the resemblance in fluorescent response

Spectroscopy of human insertion site, bone and cartilage was performed to assess its spectral response (*n* = 3). Spectral responses between the human tissue and bovine tissue was similar, which allowed a similar imaging and unmixing strategy to be used for both sample types, (Fig. 2).

### ACL insertion site imaging based on spectral unmixing

**Fig. 3:**
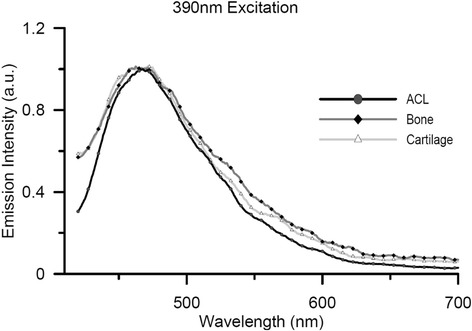
Normalized bovine emission curves of ACL, Bone and Cartilage at 390 nm excitation. Note that the curve between ACL and Bone deviates around 525 nm

**Fig. 4:**
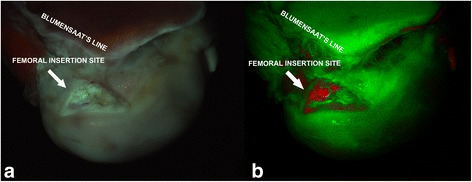
**a**: Bovine lateral femoral condyle in grayscale. ACL is indicated by arrow. It has to be noted that the insertion site can be easily seen in this macroscopic and sagittal section, however this is difficult when viewed arthroscopically. **b**: False color image of the same bovine lateral femoral condyle with femoral insertion site. Notice how the other ligaments are also enhanced by the method

The measurements with the fluorospectrometer were used to find the most optimal method to enhance the contrast between the tissues as described in the previous paragraph. It followed from these measurements that direct excitation with 280 nm can be used to obtain the largest peak difference in emission wavelength. Excitation with 330–340 nm can be used to obtain the largest intensity difference between the tissues. Because both approaches are technically not practicable, as elaborated in the discussion, we decided to focus on the small but distinctive difference in emission spectra above 525 nm between the insertion site and its surroundings for a fixed excitation wavelength of 390 ± 20 nm (Fig. 3). We mean with a distinctive difference in emission spectra above 525 nm that the contribution of the ACL is smaller than the contribution of bone and cartilage. While the opposite is true for under the 525 nm. Such difference can be utilized to obtain a unique ACL insertion site image with spectral unmixing and displayed in pseudocolors. Typical contrasting results of the knee after spectral unmixing with an excitation of 390 ± 20 nm are shown in Fig. 4b. The femoral insertion site is shown in bright red while the bony and cartilage surrounding seen in a contrasting green.

### Validation of spectral unmixing in bovine knees

The accuracy of the spectral unmixing in discerning the femoral ACL insertion site from the background was assessed by three independent observers who draw the contour of the femoral insertion site in the images (*n* = 10). These contours were then compared to the segmented images from the spectral unmixing routine by creating binary images of the ACL insertion site. The Dice coefficient for observer 1, 2 and 3 between the unmixed and the white light images was 0.809, 0.831 and 0.874 respectively (Table 1). The observers preferred annotating in the unmixed autofluorescence images. The Dice coefficient for interobserver variability for the white light images for observer 1, 2 and 3 was 0.844, 0.865, and 0.836 respectively (Table 2). The Dice coefficient for interobserver variability for the unmixed images for observer 1, 2 and 3 was 0.796, 0.853, and 0.832 respectively (Table 3). The Dice coefficients of the white light images vs unmixed images and the white light interobserver Dice were not statistically different (*p* = 0.57). The same holds for white light images vs unmixed images and the unmixed interobserver Dice (*p* = 0.56) and the white light images interobserver vs unmixed images interobserver Dice (*p* = 0.20).

**Table 1:** Dice coefficient spectral unmixed vs white light

Knee	Observer 1	Observer 2	Observer 3
1	0.878	0.915	0.900
2	0.875	0.899	0.860
3	0.834	0.722	0.760
4	0.737	0.813	0.727
5	0.892	0.831	0.938
6	0.673	0.761	0.911
7	0.721	0.725	0.887
8	0.888	0.903	0.941
9	0.754	0.872	0.913
10	0.836	0.868	0.906
mean	0.809 ± 0.08	0.831 ± 0.07	0.874 ± 0.07

**Table 2:** Dice coefficient Interobserver variability white light

Knee	Observer 1	Observer 2	Observer 3
1	0.867	0.905	0.900
2	0.858	0.872	0.864
3	0.859	0.848	0.810
4	0.872	0.892	0.865
5	0.889	0.890	0.864
6	0.744	0.796	0.694
7	0.713	0.789	0.747
8	0.880	0.890	0.847
9	0.871	0.890	0.869
10	0.882	0.882	0.896
Mean	0,844 ± 0.06	0,865 ± 0.04	0.836 ± 0.06

**Table 3:** Dice coefficient Interobserver variability spectral unmixed

Knee	Observer 1	Observer 2	Observer 3
1	0.913	0.914	0.921
2	0.842	0.892	0.870
3	0.699	0.793	0.805
4	0.670	0.776	0.733
5	0.904	0.926	0.903
6	0.864	0.901	0.863
7	0.778	0.809	0.799
8	0.809	0.865	0.816
9	0.734	0.810	0.784
10	0.743	0.843	0.827
Mean	0.796 ± 0.08	0.853 ± 0.05	0.832 ± 0.05

### Real-time spectral unmixing images and video with prototype of Fluorescence Arthroscope

**Fig. 5:**
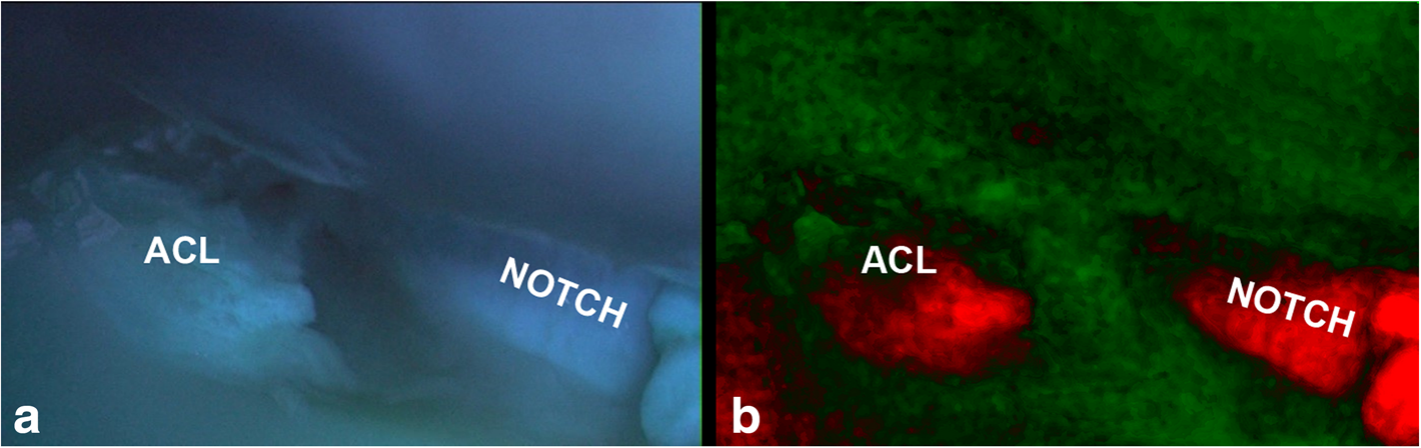
Inferior view of femoral condyles in bovine knee. **a** color image of the fluorescent response as directly captured by the digital camera. Notice the subtle differences between the ACL site (bluish/greenish) and its surroundings. **b** Real-time unmixed result of the color image left, with red the ACL and part of PCL and in green the background composed of the cartilage and bone. Fibrocartilaged notch (typical for bovine knees) also reveals in red

The knees were excited with 390 ± 20 nm and the fluorescent video was captured and processed real time (Fig. 5 and see Additional file 1). The video shows a sharp bordered femoral insertion site in bright red with a contrasting background in green. The clear image quality remained during the imaging and no decrease of the autofluorescence (photo bleaching) and image quality was noted.

### Proof of concept: ex vivo human Autofluorescence Arthroscopy

**Fig. 6:**
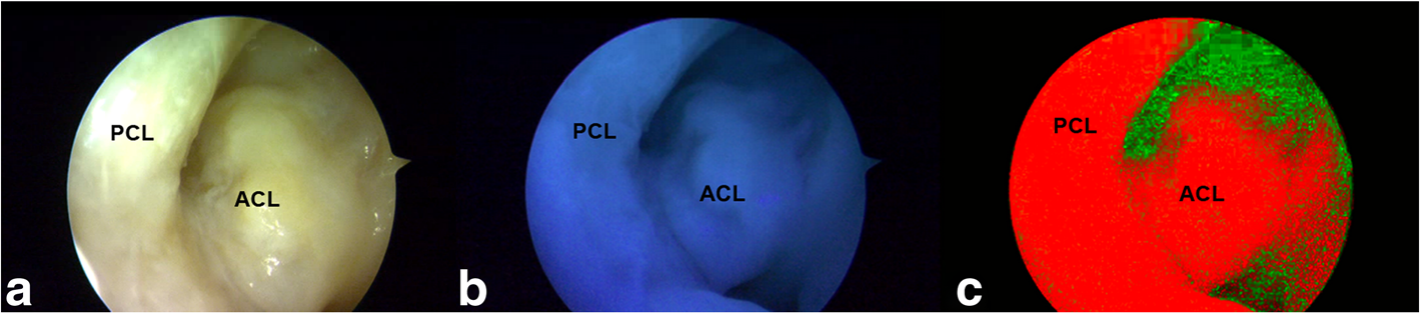
Arthroscopic view of a cadaveric human femoral insertion site. Consecutively shown in white light mode (**a**), fluorescence mode (**b**), and spectral unmixed mode (**c**)

The next step was to adapt the AA prototype for inclusion in a commercial available endoscopic system to visualize the insertion site as in the clinic. The view through an arthroscope of a cadaveric human insertion site was consecutively shown in white light mode, fluorescence mode and spectral unmixed mode (Figs. 6a, b, and c). From all these modes, the spectral unmix mode clearly shows the most optimal contrast between the insertion site and its background. The structure on the left of the ACL that has the same color and intensity is the posterior cruciate ligament.

## Discussion

In this study, we have shown that the spectral response between the insertion site, surrounding bone and cartilage differs. Thanks to this difference and with the aid of fluorescence imaging combined with spectral unmixing, the femoral insertion site could be visualized in contrasting pseudo-colors. The accuracy of localization of the femoral insertion site with fluorescence imaging is high and comparable to localization of the femoral insertion site during open surgery. As a proof of concept, we have developed a prototype of the Autofluorescence Imaging system which was successfully tested in an *ex vivo* setting, including the 30° degrees arthroscope. The AFI demonstrated to be robust and easily integrated into a current commercial white light endoscopic systems. Additional improvements and *in vivo* studies are needed prior to clinical use.

Excitation with 330–340 nm yields the largest intensity difference between the tissues. However, 330 nm falls within the UV range and may be harmful with prolonged exposure. Excitation with 280 nm to obtain the largest difference in emission peak was for the same reasons not feasible. Excitation with a longer and safe wavelength (390 nm) showed autofluorescence of the insertion site, surrounding bone and cartilage. Though the relative intensity of the insertion site was not enough to clearly discern it from the background by eye. Also, using a filter to block the autofluorescence of the background was not to be effective to enhance the contrast between these tissues. Spectral unmixing with excitation at 390 nm and emission based on 520 nm and 620 nm images did enable accurate and subject specific visualization of the native femoral insertion site.

Since 2001, researchers have reported their concern about the accuracy of placing the graft with the transtibial technique and thus its ability to restore knee stability (Arnold et al. 2001; Bedi et al. 2010). According to a recent meta-analysis, only 41% of patients have reported their reconstructed knee as normal (Biau et al. 2007). Due to these suboptimal results, there is a paradigm shift ongoing from the “transtibial” towards the “anatomic” ACL reconstruction technique (Yasuda et al. 2004; Mae et al. 2010; van Eck et al. 2010; Chechik et al. 2013). The “anatomic” ACL reconstruction aims to place the graft at the native insertion sites by relying on arthroscopically visualizing anatomical landmarks such as the posterior wall, cartilage edge, the lateral intercondylar ridge, and bifurcate ridge to locate the native insertion sites (Yasuda et al. 2004; Farrow et al. 2007; Ferretti et al. 2007; Mae et al. 2010; van Eck et al. 2010; Ziegler et al. 2011). This method, though is time consuming and demanding due to the visual limitations with the current white light endoscopic systems, careful dissection, biological variability, and additional visual impairment in the injured and degenerated joint. Hence, the “transtibial” technique was introduced to circumvent these difficulties. Several aides for arthroscopic visualization have been proposed to assist anatomic ACL reconstructions, including intraoperative fluoroscopic (Larson et al. 1995; Klos et al. 1998; Kawakami et al. 2012; Moloney et al. 2013) and computer-aided navigation systems (Dessenne et al. 1995; Burkart et al. 2001; Zaffagnini et al. 2010). The use of these systems, however are often criticized for being expensive, time consuming, and ionizing hazards (Rivkin and Liebergall 2009; Kasten et al. 2010). The AFI, however does not have these drawbacks. Additional advantages of the AFI include: real time, tissue specific, subject specific imaging, as well as easy, fast and low cost. The AFI system was developed to image autofluorescence of collagen and injury- or degeneration related spectral variations due to changes in collagen content or distribution. Apart from imaging the ACL, examples in which the use of the AFI can be valuable include, but are not limited to: posterior cruciate ligament reconstruction, partial meniscectomy, (preventive) meniscal repair, glenoid repair, and labrum repair. Also, bony lesions causing impingement (subacromial, hip or ankle) and bone tumors may be visualized. Furthermore, degeneration of other musculoskeletal tissues can be easily detected. Thus the AFI may also be used diagnostically.

### Limitations

A limitation of our study is that the AFI has not yet been tested in patients. The questions whether the femoral remnant is still present after rupture and whether the used excitation wavelength is safe, are legitimate. Literature has reported that the femoral remnant is still present in 98% of the patients and is collagenous (Lo et al. 1999; Wittstein et al. 2009). Hence, orthopedic surgeons routinely use a shaver to remove the femoral and tibial remnant. Future studies need to assess photo toxicity of excitation wavelengths in violet (390 nm-405 nm) to the tissues within the joints. Though, previous applications within the urology gastro-enterology and abdominal surgery have shown that the illumination with fluorescence excitation wavelengths in violet (390 nm-405 nm) is safe and harmless (Schmidbauer et al. 2004). Additionally, excitation will be short in duration (seconds). The use of fresh bovine knees and fresh-frozen cadaveric human knees have demonstrated its robustness. Therefore, we have the confidence that the native insertion site will be visible in patients. Another concern was that the image quality of the cadaveric bovine knee had a better SNR than the cadaveric human knees. A reason for this difference can be explained by the difference in freshness and storage of the samples. The bovine knees were fresh and never frozen while the human knees were frozen and stored for at least a year. Literature has shown that the autofluoresence of collageneous tissue decreases with tissue freshness and with freezing-thaw cycles (Palmer et al. 2002).

## Conclusion

In conclusion, we have shown that the femoral insertion site can be accurately visualized with autofluorescence imaging combined with spectral unmixing. The prototype of the AFI demonstrates the capability to real-time and subject specific visualize the femoral insertion site. Furthermore, the AFI system may facilitate arthroscopic procedures in other joints and may be used as a diagnostic tool during a fluorescence arthroscopy.

## Additional file

Additional file 1: Real-time spectral unmixing of lateral femoral bovine condyle. Excited with 390 ± 20 nm. Femoral ACL insertion site is shown in red. (WMV 8203 kb)
